# A Prospective Observational Study on Gastric Endoscopic Submucosal Dissection under Continuous Administration of Antithrombotic Agents

**DOI:** 10.3390/jcm13071886

**Published:** 2024-03-25

**Authors:** Daisuke Kawai, Masaya Iwamuro, Ryuta Takenaka, Taisuke Obata, Takashi Yamamoto, Shoichiro Hirata, Ko Miura, Koji Takemoto, Hirofumi Tsugeno, Shigeatsu Fujiki

**Affiliations:** 1 Department of Gastroenterology, Tsuyama Chuo Hospital, Tsuyama 708-0841, Japan; daicawai@yahoo.co.jp (D.K.); rtakenak@gmail.com (R.T.);; 2 Department of Gastroenterology and Hepatology, Okayama University Graduate School of Medicine, Dentistry, and Pharmaceutical Sciences, Okayama 700-0082, Japan

**Keywords:** endoscopic submucosal dissection, antithrombotic agents, thienopyridine, gastric tumor, postoperative bleeding, delayed bleeding

## Abstract

**Background**: This study aimed to assess the completion rate and postoperative bleeding incidence of endoscopic submucosal dissection (ESD) for gastric tumors under continuous antithrombotic therapy. **Methods**: A prospective observational study was conducted including 88 patients with 100 gastric lesions who underwent gastric endoscopic submucosal dissection (ESD) and received continuous antithrombotic therapy. Additionally, retrospective data on gastric ESD in 479 patients with 534 lesions who did not receive antithrombotic therapy were collected for comparison. **Results**: The en bloc resection rates (100% in the continuous antithrombotic therapy group vs. 100% in the non-antithrombotic therapy group) and complete resection rates (97.0% vs. 96.3%, respectively) were high and comparable between the groups. No significant differences were found in the specimen size or procedure time. Perforation rates were low (0% vs. 2.3%, respectively) and were not significantly different between the groups. However, postoperative bleeding occurred significantly more frequently in the continuous antithrombotic therapy group (10.2% vs. 4.2%, respectively) than in the non-antithrombotic therapy group. The subgroup analysis revealed a higher incidence of postoperative bleeding in patients receiving thienopyridine derivatives. **Conclusions**: Continuous administration of antithrombotic agents, especially thienopyridines, increased the risk of postprocedural hemorrhage following gastric ESD. These findings support the need for careful consideration of pharamcological management before ESD, aligning with the current guidelines.

## 1. Introduction

Gastric adenocarcinoma, a malignancy originating from the stomach’s epithelial lining, ranks as the third most prevalent cause of cancer-related mortality worldwide [1,2]. Traditionally, surgical interventions are reserved for cases presenting with an increased likelihood of lymph node metastasis or those diagnosed with submucosal invasive gastric cancer. In contrast, endoscopic submucosal dissection (ESD), an advanced endoscopic technique, has emerged as a preferred curative strategy for early-stage gastric cancers devoid of these ominous prognostic indicators. ESD entails the meticulous removal of the mucosal and submucosal tissues utilizing specialized endoscopic equipment, offering a minimally invasive alternative to conventional surgical approaches. This innovative procedure boasts high rates of efficacy and safety, making it an increasingly favored option in the management of early gastric malignancies. In Japan, early gastric cancer represents a significant proportion, ranging from 15% to 57%, of all gastric cancer cases [3,4]. This notable prevalence underscores the critical need for effective treatment strategies, with endoscopic resection established as a cornerstone in the management of these lesions [5,6]. Among various endoscopic resection techniques, ESD distinguishes itself by demonstrating superior performance metrics, including higher rates of en bloc resection and lower incidences of local recurrence compared to conventional endoscopic mucosal resection [7,8,9]. However, it is essential to acknowledge that despite these advantages, ESD carries inherent risks, with serious adverse events such as postoperative bleeding among the potential complications. Multiple reports have indicated that antiplatelet agents and anticoagulants increase the incidence of postoperative bleeding [10,11], and the discontinuation of these agents poses a risk of thromboembolism. In addition, a previous report suggested that the continuous administration of antiplatelet agents is not independently and significantly correlated with postoperative bleeding. Currently, insufficient data exist concerning the safety of the continuous use of antithrombotic agents during gastric ESD. Thus, we conducted a prospective observational study to evaluate the impact of the continuous administration of antithrombotic agents, focusing on the completion rate and incidence of postoperative bleeding following ESD for gastric tumors. Furthermore, to investigate whether the continuous oral administration of antithrombotic agents increases postoperative bleeding, we retrospectively collected and compared data on gastric ESD performed during the same period in patients who did not receive antithrombotic therapy.

## 2. Materials and Methods

### 2.1. Prospective Observational Study on Gastric ESD in Patients Undergoing Continuous Antithrombotic Therapy

A prospective observational study was conducted at a single hospital in Japan from November 2013 to March 2019, focusing on patients aged ≥20 years diagnosed with or suspected to have early gastric cancer and presenting a negligible risk for lymph-node metastasis. This study included individuals receiving antithrombotic agents, including antiplatelet agents (e.g., aspirin, thienopyridine derivatives, or cilostazol) and antithrombotic agents (e.g., warfarin or direct oral anticoagulants). ESD was indicated for (i) differentiated mucosal cancer lesions without ulcers, regardless of size, (ii) differentiated mucosal cancer lesions ≤3 cm with ulcers, (iii) undifferentiated mucosal cancer lesions ≤2 cm without ulcers, and (iv) adenoma suspected of containing a mucosal cancer component. For all lesions, lymph-node metastasis-free and distant metastasis-free conditions were confirmed using preoperative computed tomography.

The study was approved by the institutional review board of our hospital on 6 February 2013 (number 153) and was conducted in accordance with the Declaration of Helsinki. Written informed consent was obtained from all patients. This study was registered with the University Hospital Medical Network Clinical Trials Registry (trial number: UMIN000012079). All authors had full access to the study data, and the final manuscript was reviewed and approved by all authors.

### 2.2. Retrospective Observational Study on Gastric ESD in Patients without Antithrombotic Therapy

To investigate whether continuous oral administration of antithrombotic agents increases postoperative bleeding, we retrospectively reviewed patients who did not receive antithrombotic agents and underwent gastric ESD between November 2013 and March 2019. The retrospective study protocol was approved by the institutional review board of our hospital (number 626) and conducted in accordance with the Declaration of Helsinki. The requirement for written informed consent was waived due to the retrospective and anonymous nature of the data.

### 2.3. ESD Procedure

ESD was performed using a high-frequency generator (VIO 300D; Erbe Elektromedizin GmbH, Tubingen, Germany). Hemostatic forceps (FD-410LR; Olympus Co. or HDB2418W; Hoya Co., Tokyo, Japan) were used in soft coagulation mode at effect 6 and 70 W output. Prophylactic coagulation was performed on the visible vessels; however, ESD ulcer closure was not performed. Administration of an intravenous proton pump inhibitor (PPI) (lansoprazole, 30 mg, twice daily) was initiated on the day of the ESD and replaced with oral PPI (lansoprazole, 30 mg, once daily) the following day. After April 2015, vonoprazan (20 mg, once daily) was used as a substitute for PPI in patients receiving continuous antithrombotic agents. The patients were discharged 7 days after the ESD, and they underwent follow-up endoscopy 8 weeks after the procedure.

In our study, postoperative bleeding, regarded as a critical endpoint, was precisely defined as the occurrence of clinically evident bleeding necessitating immediate intervention, such as emergency endoscopic hemostasis and/or blood transfusion, coupled with a decrease in hemoglobin levels exceeding 2 g/dL.

### 2.4. Statistical Analysis

Differences between the two groups were determined using the chi-squared test or Fisher’s exact test for discontinuous variables and the Mann–Whitney U test for continuous variables. Statistical analyses were performed using the JMP software package (version 13; SAS Institute, Cary, NC, USA). Statistical significance was set at values of *p* < 0.05.

## 3. Results

**Figure 1:**
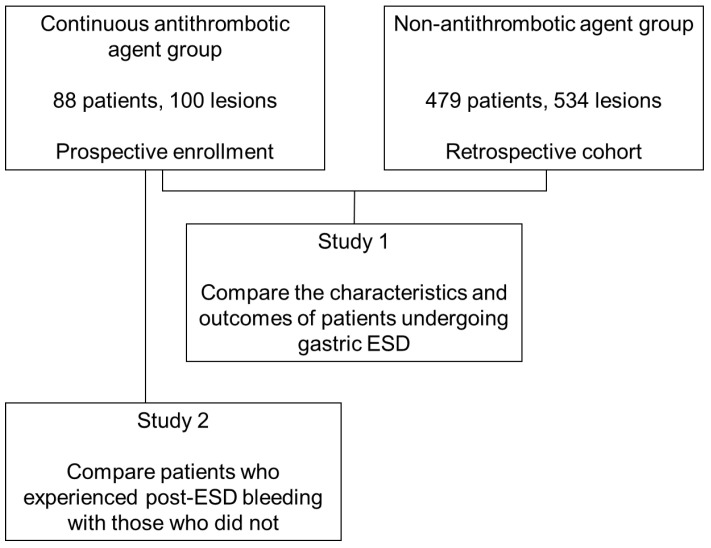
Study flowchart. ESD, endoscopic submucosal dissection.

Between November 2013 and March 2019, 88 patients with 100 gastric lesions, who received antithrombotic drugs, were enrolled in this prospective study (Figure 1). In terms of diagnostic clues for gastric lesions among 88 cases, two patients presented with anemia, and two patients tested positive for fecal occult blood. Among the two patients with anemia, one (Case No. 1) exhibited mild, normocytic normochromic anemia and revealed no lesions causing bleeding during esophagogastroduodenoscopy. No evidence of bleeding was observed from gastric cancer as well. In another patient (Case No. 2), oozing was observed from the surface of a gastric cancer, consistent with the etiology of anemia. One patient with fecal occult blood (Case No. 3) displayed bleeding gastric ulcers unrelated to gastric cancers, necessitating hemostatic intervention via esophagogastroduodenoscopy. The remaining case (Case No. 4) showed no lesions indicative of fecal occult blood on esophagogastroduodenoscopy and CT scans. Bleeding from gastric cancer was also absent.

ESD was performed in all patients, and no perforations occurred during ESD. Antiplatelet agents were administered in 72% of patients. Aspirin was administered to 35 patients, whereas cilostazole and thienopyridine derivatives were administered to 17. Only six patients received dual antiplatelet therapy. During the same period, gastric ESD was performed in 479 patients with 534 lesions who were not receiving antithrombotic agents. We retrospectively collected and analyzed data from these patients.

The clinical characteristics of the patients are summarized in Table 1. No significant difference in sex distribution was observed between the two groups (male: 70% vs. 73% in the continuous antithrombotic therapy and non-antithrombotic therapy groups, respectively). However, the age of patients was higher in the continuous antithrombotic therapy group than in the non-antithrombotic therapy group (median, 79 years vs. 74 years, respectively; *p* < 0.001). The prevalence of comorbidities associated with antithrombotic therapy including hypertension (68% vs. 56%, *p* = 0.033), cerebrovascular disease (53% vs. 3%, *p* < 0.001), ischemic heart disease (25% vs. 4%, *p* < 0.001), and atrial fibrillation (27% vs. 0.2%, *p* < 0.001) was higher in the continuous antithrombotic therapy group than in the non-antithrombotic therapy group. Although not statistically significant, alcohol intake was less frequent in the continuous antithrombotic agent group than in the non-antithrombotic agent group (28% vs. 38%, respectively; *p* = 0.074). Lansoprazole was administered to all patients in the non-antithrombotic agent group as an acid-suppressive agent following gastric ESD, whereas in the continuous antithrombotic agent group, 30% of the patients received lansoprazole and 70% received vonoprazan (*p* < 0.001).

The technical and pathological results of ESD are shown in Table 2. En bloc resection was performed in all patients, with a complete resection rate of 97.0% in the continuous antithrombotic agent group and 96.3% in the non-antithrombotic agent group, showing no statistical difference. No significant differences in specimen size (median, 35.5 mm vs. 35.0 mm) or procedure time (median, 71.5 min vs. 75.0 min) was observed between the continuous antithrombotic therapy and non-antithrombotic therapy groups, respectively. No patients in the continuous antithrombotic agent group had perforation during or after ESD, whereas 2.3% of patients in the non-antithrombotic agent group had perforations (*p* = 0.151). Postoperative bleeding was noted in 10.2% of patients in the continuous antithrombotic agent group, which was significantly more frequent than that of patients in the non-antithrombotic agent group (*p* = 0.018). None of the patients developed cardiocerebrovascular disease during the study period.

Table 3 shows the incidence of postoperative bleeding for each antiplatelet agent. Among antiplatelet agents, post-ESD bleeding was most frequent in patients taking thienopyridine derivatives (29.4%), followed by aspirin (8.6%) and cilostazol (5.9%). We further conducted a sub-analysis comparing groups with and without postoperative bleeding among patients receiving antithrombotic agents. In the postoperative bleeding group, 56% of the patients were receiving thienopyridine derivatives, which was a significantly higher proportion compared with that in the no-bleeding group (15%, *p* = 0.012) (Table 4). No statistically significant differences were observed between the two groups for any other variables.

## 4. Discussion

In the present study, en bloc resection was performed in all patients, exhibiting a complete resection rate of 97.0% observed in the continuous antithrombotic therapy group. Additionally, postprocedural bleeding following gastric ESD occurred in 10.2% of patients with continued use of antithrombotic agents. To our knowledge, this is the first prospective observational investigation of gastric ESD performed while continuing antithrombotic agent therapy. Furthermore, it highlights an elevated risk of post-ESD bleeding associated with thienopyridine use among all antithrombotic agents. Although data for the non-antithrombotic agent group were retrospectively collected, the incidence of post-ESD bleeding was higher in the continuous antithrombotic therapy group than in the non-antithrombotic therapy group. These findings emphasize the need for caution regarding the increased risk of postoperative bleeding when continuing antithrombotic agents, particularly in patients receiving thienopyridine therapy, suggesting the need for alternative agents or temporary discontinuation of antithrombotic therapy in this subset of patients.

Several studies have highlighted the increased risk of postoperative bleeding in patients undergoing gastric ESD while receiving antithrombotic therapy. Gotoda et al. found that dual antiplatelet therapy and heparin replacement are associated with this risk [12]. Matsumura et al. identified a large specimen size, heparin replacement, and chronic kidney disease requiring hemodialysis as risk factors for early and delayed postoperative bleeding [13]. Sato et al. further emphasized the risk of postoperative bleeding in patients receiving antithrombotic therapy, particularly in those receiving heparin bridging therapy and dual antiplatelet therapy [14]. Ueki et al. found that interrupted antithrombotic therapy and prolonged ESD procedure times are associated with early and delayed postoperative bleeding, respectively [15].

A number of retrospective studies have suggested that continued use of antiplatelet agents may increase the risk of post-ESD bleeding. Lim et al. investigated a cohort of 1591 subjects, 274 of whom were taking antiplatelets, with 102 discontinuing them for 7 days or more before gastric ESD [16]. Although continuous administration of antiplatelets was not independently associated with a significant increase in bleeding after ESD, the incidence of post-ESD bleeding was higher among those who continued to use antiplatelets (11.6%) compared to those who discontinued them (5.9%) or those who were not taking antiplatelets (5.2%). Igarashi et al. reported a similar incidence of 9.3% in the group continuing antithrombotic therapy, compared to 4.2% in the group not receiving antithrombotic therapy [17]. They further noted that the incidence of post-gastric ESD bleeding was comparable in patients on antithrombotic therapy with heparin bridge therapy (replaced with heparin, 10.8%) and those who ceased antithrombotic therapy (9.4%). Oh et al. examined 215 patients who underwent gastric ESD [18]. Among these, 161 patients received single-agent antiplatelet therapy (94 on aspirin, 56 on thienopyridine, and 11 on alternative agents), while 51 were administered dual therapy, and 3 were subjected to triple antiplatelet regimens. The incidence of bleeding was 15.4% in the continuous aspirin group, 11.8% in the aspirin cessation group, 7.1% in the continuous thienopyridine group, and 0% in the thienopyridine cessation group. Harada et al. reported that the post-gastric ESD bleeding rate was 15.8% in patients receiving continuous administration of antiplatelets, compared to 8.5% in patients who discontinued antiplatelet therapy [19]. According to a recent nationwide, multicenter retrospective study conducted in Japan involving a total of 9736 patients, the post-gastric ESD bleeding rates were 9.3% for the continuous administration of aspirin, 5.3% for discontinued aspirin therapy, 19.0% for continuous thienopyridine therapy, and 14.1% for discontinued thienopyridine therapy [20]. These findings underscore the need for careful monitoring and management of bleeding risk in patients undergoing gastric ESD while receiving antithrombotic therapy.

In Japan and Western countries, guidelines have been published that describe both the risk of gastrointestinal bleeding associated with continuing antithrombotic drugs and the risk of thromboembolism with the temporary discontinuation of this therapy [21,22,23]. According to these guidelines, endoscopic biopsy and endoscopic procedures with a low risk of bleeding, such as balloon endoscopy, marking clip, tattooing, pancreatic duct, and bile duct stenting, and endoscopic papillary balloon dilation, can be performed without discontinuing the antithrombotic drug. However, temporary cessation of antithrombotic drugs is still recommended for endoscopic treatments with a high risk of bleeding, such as polypectomy, ESD, and endoscopic papillotomy. Currently, data supporting the efficacy of these tentative guidelines are insufficient. Hence, evaluating whether the continuous administration of antithrombotic agents influences the efficacy of ESD and increases the risk of post-ESD bleeding is important. In this prospective cohort study, ESD was performed without the cessation of antithrombotic agents, and the complete resection rate for ESD was 97.0%, and post-ESD bleeding occurred in 10.2% of the patients.

The incidence of post-ESD bleeding has been retrospectively reported to be 1.8–15.6% [10,11,13,17,24,25,26,27,28,29,30,31,32,33]. Risk factors for post-ESD bleeding include age [29], hemodialysis [13,30], longer operation time [29,31,33], tumor size [28], resection size [11,13,17,24,31,32], tumor location [10,27,28,31], scar lesion [31], recurrent lesion [28], macroscopic type [28], and administration of antithrombotic agents [11,33]. In this prospective cohort study, only the continuous administration of thienopyridine derivatives showed a significant association with the occurrence of postoperative bleeding, while under continuous administration of antithrombotic agents. Ono et al. reported a significantly higher incidence of postoperative bleeding in patients receiving continuous thienopyridine derivatives and multiple antithrombotic agents than in those not receiving any medication [34]. In a study involving patients who underwent gastric ESD with aspirin withdrawal and continued thienopyridine administration after coronary artery stent placement, the postoperative bleeding rate was reported to be 20% [35]. Tounou et al. reported a postoperative bleeding rate of 35.5% in the aspirin and thienopyridine-derivative administration group [36]. A recent nationwide, multicenter retrospective study conducted in Japan also unveiled that among patients undergoing gastric ESD, the incidence of post-procedural bleeding was notably elevated in those receiving continuous thienopyridine therapy (19.0%), as opposed to individuals with discontinued thienopyridine therapy (14.1%), continuous aspirin administration (9.3%), discontinued aspirin therapy (5.3%), cessation of cilostazol (2.1%), and continuous cilostazol use (1.8%) [20]. The results of the present study, together with those of the existing literature, suggest that thienopyridine is associated with a higher risk of bleeding than other antiplatelet agents. As described in the Japanese guidelines [37], switching thienopyridine to another antiplatelet agent (e.g., aspirin or cilostazol) is considered appropriate for preventing postoperative bleeding.

Our study underscores several significant limitations that necessitate thorough consideration. First, this investigation was carried out exclusively at a singular medical center, which inherently restricts the broader applicability of our findings due to the relatively confined sample size involved in our study cohort. Additionally, within the subset of patients receiving continuous antithrombotic agents, there was a notable transition observed in oral acid-suppressive therapy, shifting from lansoprazole to vonoprazan in the study period. While the incidence rates of postoperative bleeding remained relatively consistent, it is plausible that this alteration may have introduced variability in clinical outcomes. Moreover, data pertaining to patients not receiving antithrombotic medications were retrospectively acquired, potentially introducing inherent biases into our analyses. Nevertheless, notwithstanding these acknowledged limitations, our study leverages prospective observational data garnered from patients undergoing gastric ESD while adhering to antithrombotic drug regimens. We posit that these insights offer valuable real-world perspectives into the clinical management of individuals navigating such therapeutic regimens.

## 5. Conclusions

In conclusion, our prospective investigation revealed that achieving outstanding en bloc resection (100%) and complete resection (97.0%) rates is feasible with the continuous administration of antithrombotic agents. Postprocedural hemorrhage following gastric ESD manifested in 10.2% of the patients in whom antithrombotic agents were consistently administered. Notably, the heightened risk of bleeding observed in patients receiving thienopyridine underscores the imperative need for medication cessation or modification prior to the procedure. This outcome aligns with the current guidelines, thereby substantiating the necessity for such interventions.

## Figures and Tables

**Table 1:** Clinical characteristics of the patients.

		Continuous Antithrombotic Agent Group 88 Patients, 100 Lesions		Non-Antithrombotic Agent Group 479 Patients, 534 Lesions		*p*-Value
Age (years), median (range)		79	(60–92)	74	(33–94)	<0.001
Sex, male, *n* (%)		62	(70)	349	(73)	0.642
Habit						
	Smoking, *n* (%)	14	(16)	91	(19)	0.493
	Alcohol consumption, *n* (%)	25	(28)	184	(38)	0.074
Comorbidities						
	Hypertension, *n* (%)	60	(68)	268	(56)	0.033
	Diabetes mellitus, *n* (%)	25	(28)	113	(24)	0.333
	Cerebrovascular disease, *n* (%)	47	(53)	15	(3)	<0.001
	Ischemic heart disease, *n* (%)	22	(25)	16	(4)	<0.001
	Atrial fibrillation, *n* (%)	24	(27)	1	(0.2)	<0.001
Antithrombotic agent						
	Antiplatelet agents, *n* (%)	63	(72)			
	Aspirin, *n* (%)	35	(40)			
	Cilostazol, *n* (%)	17	(19)			
	Thienopyridine derivatives, *n* (%)	17	(19)			
	Anticoagulant agent	27	(31)			
	Warfarin, *n* (%)	11	(13)			
	Apixaban, *n* (%)	9	(10)			
	Rivaroxaban, *n* (%)	4	(5)			
	Dabigatran, *n* (%)	3	(3)			
Number of antithrombotic agents						
	Single, *n* (%)	80	(91)			
	Multiple, *n* (%)	8	(9)			
	Dual antiplatelet agents	6	(7)			
	Aintiplatelet plus anticoagulant	2	(2)			
Lesion size (mm), median (range)		12	(2–140)	12	(1–64)	0.343
Number of lesions						0.731
	Single, *n* (%)	76	(86)	420	(88)	
	Multiple, *n* (%)	12	(14)	59	(12)	
Acid-suppressive agent						<0.001
	Lansoprazole	26	(30)	479	(100)	
	Vonoprazan	62	(70)	0	0	

**Table 2:** Technical and pathological results of endoscopic submucosal dissection.

	Continuous Antithrombotic Agent Group 88 Patients, 100 Lesions		Non-Antithrombotic Agent Group 479 Patients, 534 Lesions		*p*-Value
En bloc resection, *n* (%)	100	(100)	534	(100)	1.000
R0 resection, *n* (%)	97	(97.0)	514	(96.3)	0.715
Specimen size (mm), median (range)	35.5	(22–150)	35.0	(17–96)	0.205
Procedure time (min), median (range)	71.5	(23–273)	75.0	(22–445)	0.876
Adverse event					
Perforation, *n* (%)	0	(0)	11	(2.3)	0.151
Postoperative bleeding, *n* (%)	9	(10.2)	20	(4.2)	0.018

**Table 3:** The incidence of postoperative bleeding for each antiplatelet agent.

	Incidence of Postoperative Bleeding (%)
Antithrombotic agents, overall	9/88 (10.2)
Antiplatelet agents	7/63 (11.1)
Aspirin	3/35 (8.6)
Cilostazol	1/17 (5.9)
Thienopyridine derivatives	5/17 (29.4)
Anticoagulant agents	2/27 (7.4)
Warfarin	0/11 (0)
Apixaban	1/9 (11.1)
Dabigatran	0/3 (0)
Rivaroxaban	1/4 (25.0)
Number of antithrombotic agent	
Multiple	2/8 (25.0)
Dual antiplatelet agents	2/6 (33.3)
Antiplatelet + anticoagulant	0/2 (0)

**Table 4:** Factors associated with postoperative bleeding in patients receiving antithrombotic agents.

	Postoperative Bleeding *n* = 9		No Postoperative Bleeding *n* = 79		*p*-Value
Age (years), median (range)	77	(69–89)	79	(60–92)	0.634
Sex, male, *n* (%)	7	(78)	55	(70)	1.000
Habit					
Smoking, *n* (%)	0	(0)	14	(18)	0.344
Alcohol consumption, *n* (%)	1	(11)	24	(30)	0.436
*Helicobacter pylori* infection status					
Positive, *n* (%)	3	(33)	25	(32)	1.000
Comorbidity					
Hypertension, *n* (%)	6	(67)	54	(68)	1.000
Diabetes mellitus, *n* (%)	2	(22)	23	(29)	1.000
Cerebrovascular disease, *n* (%)	6	(67)	41	(52)	0.494
Ischemic heart disease, *n* (%)	0	(0)	22	(28)	0.105
Atrial fibrillation, *n* (%)	2	(22)	22	(28)	1.000
Antithrombotic agent					
Antiplatelet agent	7	(78)	56	(71)	1.000
Aspirin, *n* (%)	3	(33)	32	(41)	1.000
Cilostazol, *n* (%)	1	(11)	16	(20)	1.000
Thienopyridine derivatives, *n* (%)	5	(56)	12	(15)	0.012
Anticoagulant agents	2	(22)	25	(32)	0.716
Warfarin, *n* (%)	0	(0)	11	(14)	0.595
Apixaban, *n* (%)	1	(11)	8	(10)	1.000
Dabigatran, *n* (%)	0	(0)	3	(4)	1.000
Rivaroxaban, *n* (%)	1	(11)	3	(4)	0.356
Number of antithrombotic agent					
Multiple, *n* (%)	2	(22)	6	(8)	0.188
Dual antiplatelet agents	2	(22)	4	(5)	0.113
Antiplatelet + anticoagulant	0	(0)	2	(3)	1.000
Location of lesion (*n* = 100)					
Upper third, *n* (%)	2	(20)	10	(11)	0.644
Middle third, *n* (%)	5	(50)	43	(48)	
Lower third, *n* (%)	3	(30)	37	(41)	
Lesion size (mm), median (range)	16	(12–40)	13	(4–140)	0.119
Number of lesions					
Multiple, *n* (%)	1	(11)	11	(14)	1.000
Acid-suppressive agent					
Vonoprazan	6	(67)	56	(71)	1.000

## Data Availability

The datasets generated and analyzed in the current study are available from the corresponding author upon reasonable request.
